# Evaluation of a city-wide physical activity pathway for people affected by cancer: the Active Everyday service

**DOI:** 10.1007/s00520-022-07560-y

**Published:** 2023-01-09

**Authors:** Liam Humphreys, Gabriella Frith, Helen Humphreys, Helen Crank, Joanne Dixey, Diana M Greenfield, Lindsey J Reece

**Affiliations:** 1 Academy of Sport and Physical Activity, Sheffield Hallam University, Collegiate Crescent Campus, Sheffield, S10 2BP UK; 2 Advanced Wellbeing Research Centre, Sheffield Hallam University, Sheffield, UK; 3 Centre for Behavioural Science and Applied Psychology, Sheffield Hallam University, Sheffield, UK; 4 Therapy Services, Sheffield Teaching Hospitals NHS Foundation Trust, Sheffield, UK; 5 Specialised Cancer Services, Sheffield Teaching Hospitals NHS Foundation Trust, Sheffield, UK; 6 Department of Oncology and Metabolism, University of Sheffield Medical School Beech Hill Road, Sheffield, UK; 7 SPRINTER Research Group, Prevention Research Collaboration, Charles Perkins centre, School of Public Health, University of Sydney, Sydney, Australia

**Keywords:** Exercise referral, Exercise, Physical activity, Cancer survivorship, Community-based

## Abstract

**Purpose:**

The primary goal of this article is to present an evaluation of a UK-based city-wide physical activity pathway for patients with a cancer diagnosis, the Active Everyday service. Active Everyday was a co-produced physical activity service for people affected by cancer. The service was underpinned by a behaviour change care pathway model developed by Macmillan Cancer Support charity.

**Methods:**

This was a retrospective evaluation assessing physical activity levels and changes to outcome measures (fatigue, perceived health, and self-efficacy) over 6 months. Each participant self-reported their levels of physical activity for the previous 7 days at three-time points: baseline (T1), at 12-week exit from the scheme (T2), and at 6-month follow-up (T3).

**Results:**

The Active Everyday service received 395 referrals, of which 252 attended a baseline assessment. Participants’ fatigue and self-efficacy improved between T1 and T2 and T1 and T3. Perceived health improved across all time points. Participant exercise levels showed significant differences between T1 and T2.

**Conclusion:**

The service, provided over 3 years, resulted in positive health and wellbeing outcomes in people affected by cancer who engaged in the service. Future services must routinely include exercise referrals/prescriptions as a standard part of care to help engage inactive individuals. Services should focus on targeted promotion to people from ethnic minority groups, and a wide socioeconomic population.

**Supplementary Information:**

The online version contains supplementary material available at 10.1007/s00520-022-07560-y.

## Introduction

In the United Kingdom (UK), over half of adults currently under the age of 65 will be diagnosed with cancer at some point in their lifetime [1]. The UK’s cancer survival rates (10 or more years) have doubled in the last 40 years [2]. Improved prognoses have created a need to tackle cancer survivors’ health issues [3], such as heart disease, osteoporosis, and chronic fatigue [4]. Cancer, its treatment, and related comorbid conditions can impact people affected by cancer (PABC) long after treatment has ended. Additionally, cardiovascular disease has emerged as the leading non-malignant cause of morbidity and mortality in cancer survivors, compounded by an ageing population; more people will survive cancer and require support to live with the potential late effects of their cancer treatment [5].

Subsequently, there is a growing need to improve the long-term care of PABC and cancer rehabilitation has a vital role to play [6]. The central principle of cancer rehabilitation is to help cancer survivors regain and improve their physical functioning within the limitations imposed by the disease and its treatment [7]. Clinical research has provided evidence that exercise is a safe and effective intervention for PABC [8]. Regular participation in moderate-to-vigorous intensity exercises, such as brisk walking or structured exercise programmes, is associated with improved survival for several cancers (e.g. breast, colorectal, and prostate) [9, 10], improved physical function [11], diminished cancer-related fatigue [12], and improved quality of life [13].

The benefits of exercise on patient health and wellbeing have led to calls for exercise to be integrated as part of standard clinical care for PABC [14, 15]. The Clinical Oncology Society of Australia (COSA) released a position statement [8] recommending that exercise be prescribed to all patients with cancer as part of their treatment regimen. The American College of Sports Medicine (ACSM) has released exercise guidelines for PABC [3], which recommends that exercise prescription be tailored to the individual. Aligned with the general population, PABC should aim for the World Health Organisation’s global physical activity guidelines [16]. The guidelines recommend that PABC engage in 150–300 min of moderate-intensity exercise, or 75–150 min of vigorous exercise, and two strength-training sessions per week [17].

Despite the well-documented benefits of exercise, PABC do not meet the recommended physical activity and exercise guidelines [18]. Approximately 10% of PABC will meet the recommended amount of activity during treatment, and approximately 22% will meet the recommendations after treatment [19]. Additionally, PABC have varying side effects from treatments and different needs. Therefore, it would be naïve to think a one-size-fits-all approach to exercise prescription would suit patients [20]. Exercise prescription for cancer patients must therefore be tailored to individual capabilities to ensure a balance of safety, inclusivity, and effectiveness [21]. Consequently, behavioural support interventions are required to ensure PABC benefit from regular exercise [22].

Translating the current evidence for physical activity for PABC into practice is a multifactorial problem [23], which includes exercise assessment, advice, referral, and engagement. Translation of physical activity behaviour change interventions into real-world settings is the third-highest ranked research priority in exercise oncology [24]. As such, the primary goal of this article is to present an evaluation of a city-wide physical activity pathway in Sheffield, UK (the Active Everyday project). We also offer reflections on the challenges to implementation and implications for practice and research to help make exercise referral a standard procedure for PABC.

## Methods

### Active Everyday service design

Sport England made a change in strategic direction following the release of the UK government Sporting Futures strategy [25], with a broadened focus strategy [25], on the role of sport and the critical role sport plays in tackling physical inactivity. The result was a nationally funded grant programme that aimed to build the evidence base for tackling physical inactivity through sport. Macmillan Cancer Support was one of the successful recipients. Sheffield, a city in the North of England, UK, was one of six Macmillan’s pilot sites for understanding how their exercise behaviour change pathway could be adapted and implemented to support all cancer patients in initiating and sustaining a physically active lifestyle [26].

Active Everyday (AE) [27] aimed to pilot the physical activity behaviour change care pathway model developed by Macmillan Cancer Support [28]. The model was based on the NHS physical activity pathway model, Let’s Get Moving [29], NICE guidance [30], and Macmillan’s insight research [26]. To achieve this, AE adhered to the fundamental principles of the pathway but also aligned with local priorities and the exercise opportunities available locally, such as exercise referral and walking groups. The AE service was embedded into the local delivery of the Recovery Package. The recovery package is a series of critical interventions that, when delivered together, can improve outcomes for PABC [31]. Additionally, a previous qualitative project conducted by the team also underpinned the design of the service [32], reinforcing this project’s evidence-based nature. The AE team included researchers, a service manager, delivery staff, and two healthcare professionals (HCP) (a consultant nurse and a physiotherapist).

### Referral process

HCPs state that the referral process for physical activity support needs to be straightforward [32]. Referrals to AE required minimal information (name, contact details, a brief description of the patient) to limit time on behalf of the HCP. Accessibility for PABC was a key consideration, and the AE team ensured that PABC could access the programme by referral from various sources. Referral sources included HCPs (consultants, clinical nurse specialists, physiotherapists), community workers (health trainers, cancer support workers), and self-referral. Health professional referrals came from the collaboration partner Sheffield Teaching Hospital (Weston Park Cancer Centre, Royal Hallamshire Hospital, and Northern General Hospital). Members of the AE team (LH and GF) met with HCPs to brief them on the service. Other AE team members (DG and JD) are HCPs and were able to promote the service within Sheffield Teaching Hospital. HCPs were provided with flyers and business cards to either make a direct referral or encourage people affected by cancer to self-refer. Posters and banners were also placed in waiting rooms and within local cancer support centres. To be referred to AE, an individual must have previously been diagnosed with cancer, be over 16 years of age, and be interested in receiving physical activity support. There was no restriction on time since an individual’s cancer diagnosis.

### Behaviour change consultation

Upon receiving a referral, a member of the AE team contacted the PABC to schedule a behaviour change consultation. The consultation was the central component of the pathway and was underpinned by motivational interviewing principles to ensure a person-centred approach [33]. The consultations were delivered face-to-face by members of the AE team (LH and GF) who are trained in motivational interviewing and experienced in delivering exercise prescriptions to people affected by cancer. Each consultation lasted approximately 1 h and included a discussion about experiences during their cancer journey, reasons for becoming more active, what activities might be suitable for them, anticipated barriers, and goal setting. Additional resources were incorporated when needed, including decisional balance to help overcome ambivalence and a weekly planner to help overcome barriers to becoming physically active. AE team members (LH and GF) regularly discussed consultations and shadowed each other’s sessions to ensure consistency of the service delivery.

At the beginning of the consultation, participants discussed the project, signed informed consent, and completed baseline questionnaires. The Macmillan cancer and physical activity standard evaluation framework determined the service outcome measures [34]. The primary outcome for the evaluation was the total number of minutes of physical activity and exercise using the Scottish Physical Activity Questionnaire (SPAQ) [35]. Secondary measures were fatigue using the FACIT [36], self-efficacy using the General Self-efficacy Questionnaire (GSE) [37], and perceived health measured using the EQ5D Visual Analogue Scale [38]. The participants received a follow-up behaviour change consultation at 12 weeks and 6-month post-baseline. Follow-up consultations were delivered either face-to-face or by telephone and included discussions about experiences since the last consultation, any changes made, barriers they have faced, and reassessment of goals. Outcome measures were also collected at the follow-up consultation.

### Signposting to physical activity

Following the consultation, the individual was signposted to the relevant exercise opportunities that existed city-wide. The AE team developed links with strategic partners across Sheffield. Strategic partners included leisure providers, professional sports clubs, and community exercise providers. Options included access to a cancer exercise specialist (gym-based), self-management (e.g. personally tailored home-based exercise), local community providers (e.g. walking groups, yoga), or sports (e.g. walking football). Twelve instructors across the city were upskilled via training as level 4 ‘cancer exercise specialist’. Level 4 refers to a ‘specialist exercise instructor’ and is endorsed by the Chartered Institute for the Management of Sport & Physical Activity (CIMSPA) [39, 40].

### Ethical approval

Sheffield Hallam University research ethics committee granted ethical approval (HWB-S&E-8). All participants referred to the scheme gave written consent for their data to be used for evaluation purposes.

### Statistical analysis

To ensure the selection of suitable statistical analysis procedures, the parametric nature of all variables was first explored within SPSS (IBM SPSS Statistics 24.0). Histograms and Q-Q plots were visually inspected, and a Shapiro-Wilk test was conducted to assess the normality of all variables. Due to the data not being normally distributed for all variables, a non-parametric test was used. Wilcoxon signed rank test was used to analyse differences in self-reported levels of exercise, fatigue, self-efficacy, and perceived health between the three measurement points (T1–T2, T1–T3, and T2–T3).

## Results

**Figure 1:**
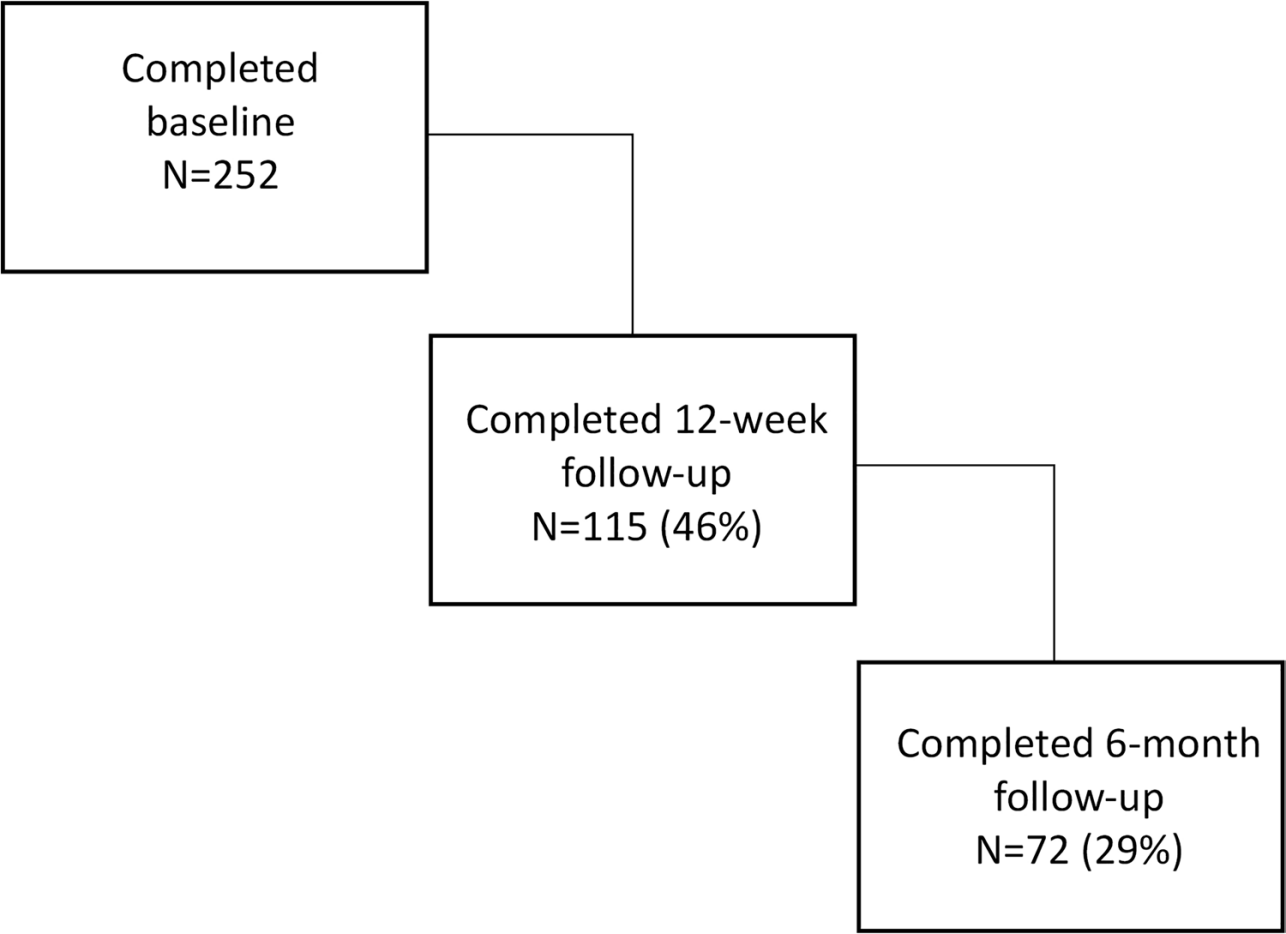
Attrition rate from the service

In total, the AE service received 395 referrals (2015 to 2018). The number of referrals to the service increased each year; in year one, 92 referrals, in year two, 142 referrals, and in year three, 161 referrals. Clinical nurse specialists (CNSs) (36%) were the top referral source, followed by self-referral (29%), and then local cancer support centres (24%). Of the 395 people referred to AE, 252 (64%) attended a baseline appointment. Figure 1 shows the attrition rates of participants through the service. All participants who provided data in T2 and T3 attended a follow-up consultation. The AE team attempted to contact service users to attend follow-up sessions. Mostly, the team could not contact people and had to report them as ‘lost to follow-up’. Reasons for not attending a follow-up appointment included lost to follow-up (40%) and able to exercise independently and, therefore, do not require more support (22%).

### Participant characteristics

Table 1 outlines the cohort’s demographic information, including age, gender, ethnicity, cancer site, stage of cancer treatment, and education level. Participants that engaged in AE were predominantly of White British ethnicity (93%). The project attracted more females (63%) than males (37%), and the most prominent cancer type was breast cancer. Many patients that engaged in the AE project were post-treatment (48%) at baseline, but many patients engaged during treatment (26%). Patients with advanced or secondary cancers also engaged in the service (18%). Service users were predominately well educated, with 66% educated to at least further education level (A-Level/BTEC).

**Table 1:** Participant demographics

	Baseline	12 weeks	6 months
Number	252	115	72
Age	57 (15.12)	57 (16.34)	59 (13.62)
Gender			
Male	93 (37%)	50 (43%)	37 (51%)
Female	159 (63%)	65 (57%)	35 (49%)
Ethnicity			
White British	234 (93%)	104 (90%)	68 (95%)
Asian-Indian and Pakistani	8 (3%)	6 (5%)	5(5%)
White — other	6 (2%)	4 (4%)	2 (3%)
Black	3 (1%)	0	0
Other	2(1%)	2 (2%)	2 (3%)
Cancer site			
Breast	102 (41%)	40 (35%)	22 (31%)
Prostate	26 (10%)	14 (12%)	10 (14%)
Lung	18 (7%)	4 (4%)	4 (6%)
Colorectal	15 (6%)	7 (6%)	4 (6%)
Head and neck	15 (6%)	10 (8%)	3 (5%)
NHL	13 (5%)	4 (4%)	2 (3%)
Other	58 (23%)	31 (27%)	24 (34%)
Cancer stage			
Treatment has been effective (recovery)	121 (48%)	61 (53%)	45 (63%)
Currently in treatment	66 (26%)	23 (20%)	11 (15%)
‘Watch and wait’ (not in active treatment)	28 (11%)	20 (17%)	10 (14%)
Cancer still present after treatment	12 (5%)	7 (3%)	4 (6%)
Unsure	18 (7%)	2 (2%)	2 (3%)
Cancer being treated again	5 (2%)	2(2%)	
Cancer not being treated	2 (1%)		
Education attainment			
Primary (pre O’ Levels or GCSE)	22 (9%)		
Secondary (Completed O’ Levels or GCSEs)	49 (21%)		
Further education (A Levels or BTEC)	59 (25%)		
Undergraduate degree or equivalent	39 (17%)		
Postgraduate degree or equivalent	32 (14%)		
Professional qualification/PhD	24 (10%)		
Other	3 (1%)		
None of the above	5 (2%)		

A large number of participants dropped out of the service after completing baseline measures and receiving a consultation. No difference was found in gender, age, baseline activity levels, or cancer stage between baseline drop-outs and participants who attended follow-up sessions.

### Types of activity

During the baseline consultation, participants were offered a menu of exercise options. Options included access to a cancer specialist exercise instructor, local community providers (e.g. walking groups, yoga), local sporting opportunities such as walking football or tailored advice about home-based exercise/self-managed exercise. Access to an appropriately trained instructor was the most desired option for the participants (41%), followed by self-management (24%).

### Changes in outcome measures

Table 2 shows the median scores at baseline and each follow-up point. Table 3 displays the differences between outcome measures over time. A total of 115 people completed the questionnaires at least twice (baseline, 3-month, and 6-month follow-up). Participant activity levels were higher than the Chief Medical Office recommendations (150 min) at baseline (median 240 min). Participant exercise levels showed a significant increase at 3 months. A Wilcoxon signed rank test showed a statistically significant change in physical activity levels (Z= −4.313, p=0.000016). Physical activity levels were maintained at 6-month follow-up, but the results were not significant (Table 3).

**Table 2:** Median scores at baseline and each follow-up point

Measure	Baseline	N	3-month follow-up	N	6-month follow-up	N
	Median (IQR)		Median (IQR)		Median (IQR)	
SPAQ (minutes)	240 (455)	252	383 (525)	115	450 (450)	75
FACIT	30.6 (16)	252	35.5 (15)	115	37.9 (13)	75
VAS	60 (30)	252	70 (50)	115	70 (20)	75
Self-efficacy	30 (7)	252	30 (5)	115	30 (4)	75

**Table 3:** Differences between outcome measures between assessment time points

Comparison	N	Wilcoxon signed rank test	
		Z	p value
SPAQ			
T1–T2	115	−4.313	.000
T1–T3	75	−.846	.398
T2–T3	69	−.268	.789
FACIT			
T1–T2	115	−4.823	.000
T1–T3	75	−3.742	.000
T2–T3	69	−1.795	.073
VAS			
T1–T2	115	−4.076	.000
T1–T3	75	−3.418	.001
T2–T3	69	−2.539	.011
Self-efficacy			
T1–T2	115	−2.500	.012
T1–T3	75	−2.320	.020
T2–T3	69	−.092	.927

IQR, interquartile range, SPAQ, Scottish Physical Activity Questionnaire, FACIT, Functional Assessment of Chronic Illness Therapy (fatigue scale), VAS, Visual Analogue Scale (perceived health)

SPAQ, Scottish Physical Activity Questionnaire, FACIT, Functional Assessment of Chronic Illness Therapy (fatigue scale), VAS, Visual Analogue Scale (perceived health)

The Wilcoxon signed rank test demonstrated a significant improvement in participant perceived fatigue levels between T1–T2 (Z=−4.823; p=0.00001) and T1–T3 (Z=−3.742; p=0.000182). No significant differences were found between 3-month and 6-month follow-up (p=.0725).

The Wilcoxon signed rank test showed a significant improvement in perceived health between all time points. Self-efficacy showed a significant difference between T1–T2 and T1–T3. No significant difference was found between T2 and T3.

## Discussion

Clinical and professional bodies continue to advocate for integrating exercise within clinical care, yet few studies have translated the evidence into practice [24]. This article presents the design, implementation, and results of AE; a person-centred physical activity service for all PABC that connected clinical care with community physical activity support. Results showed significant improvements in physical activity, fatigue, self-efficacy, and perceived health. Challenges from service include the high drop-out rate and lack of diversity in referred individuals. These findings highlight the potential for a service such as AE and the need for a transparent, evidence-based critique of its key insights.

### Referrals

The total number of referrals was low compared to the number of PABC in Sheffield, where approximately 17,000 people are living with cancer [41] and over 3000 people are diagnosed with cancer per year [42]. Possible explanations for the low referral rate are that physical activity promotion is not part of routine care or a high priority for HCPs [32, 43]. Despite recognising the importance of physical activity, many HCPs are reluctant to promote physical activity to patients [44]. HCPs often feel that physical activity is inappropriate for some cancer patients or assume that patients do not want to talk about it [45].

Greater than 80% of PABC saw lifestyle advice as helpful and believed that doctors had a duty to provide it. [46] Previous research estimated that 21% of patients received no physical activity advice, 59% received basic advice, and 20% received in-depth advice [47]. HCPs have reported several barriers that prevent them from discussing exercise with their patients. These include a lack of confidence to discuss exercise, a lack of time during consultations, and a lack of trust in schemes available to their patients [15, 32, 48]. The increase in referrals each year suggests that HCPs’ confidence in AE increased as the service became established.

The highest referral source for the AE project was the CNSs, with other HCPs (GPs, hospital doctors, and physiotherapists) referring very few patients. However, nurses tend to have the most knowledge of exercise guidelines compared to other HCPs [49]. Additionally, due to the greater focus on survivorship within cancer care, CNSs now regard physical activity promotion as part of their role [50].

AE aimed to create a genuine ‘patient-centred’ approach that puts patients in control of their health and not merely recipients [51]. A crucial part of the patient-centred approach was to empower patients to self-manage their condition by encouraging, enabling, and supporting them to take responsibility for managing their condition and increase their autonomy [52]. Self-referral into AE allowed patients to take the initiative for improving their wellbeing. Allowing patients to refer themselves proved popular. It is to be expected that HCPs initiated the discussion of exercise and then encouraged patients to contact the service.

### Exercise options and training/experience of exercise staff

The AE team developed links with current opportunities across Sheffield to create a ‘menu of options’ for participants. The choices allowed the service users access to exercise options that fit their needs and preferences. Most individuals selected access to a cancer exercise specialist as their preferred option. Qualified exercise professionals are central to any pathway for PABC [53]. PABC require reassurance that they are being monitored by an experienced professional [32] as they have concerns about their safety when engaging in exercise [54]. Fear of exacerbating pre-existing treatment-related symptoms (e.g. worsening fatigue) has been reported as a significant barrier [55–57]. The level 4 cancer specialist instructors provided a quality standard that reassured HCPs and patients of the competencies of staff receiving referrals [32].

Although access to trained staff was the most selected option, participants selected a wide range of opportunities. Services to promote activity for PABC should incorporate supervised and independent options [32]. AE service users seemed to appreciate the menu of options. For example, self-managed physical activity options were the second most favourable option. Costs of gym memberships have previously been identified as a barrier for people affected by cancer [58–60], and home-based programmes provide a no-cost option.

### Changes in outcome measures

The evaluation measures showed a range of benefits, including increased physical activity levels, reduced fatigue, and improved perceived health and self-efficacy. Most of the significant results were compared with baseline measures (T1–T2 and T1–T3). Only perceived health showed significant improvements from T2 to T3. The improvement in fatigue and perceived health are consistent with previous research demonstrating that exercise programmes can have beneficial effects on these outcomes [61]. There is a high correlation between depression and fatigue in cancer patients [62]. Low self-reported perceived health scores are predictive of depressive symptoms [63]. Cancer-related fatigue is one of the most prevalent and distressing syndromes among cancer patients [12]. Fatigue impacts cancer patients’ overall health because of its disruptive interaction with social and functional quality of life domains [12]. Participants’ perceived health ratings and fatigue improved after engaging in the service. This highlights the potential value of patients having access to a service such as AE.

Compatible with other exercise referral schemes, the AE service resulted in increased physical activity levels [64]. At 6-month follow-up, service users maintained the increase in exercise. However, the data showed that the service users averaged over 240 min of exercise at baseline. This finding is inconsistent with previous research, which states that only 34% of PABC meet the recommended amount of activity [65]. A possible explanation for the high baseline exercise levels could be the well-documented weaknesses of self-report questionnaires [66]. The SPAQ has potential errors from including light intensity exercise, overestimating walking, and active housework [66]. Exercise intensities should be clearly explained to participants in future services, and accelerometers should be used to gather objective physical activity levels.

The AE service was designed to be accessible for anyone affected by cancer who desired support. Participants were not excluded due to their baseline activity levels. Therefore, it is conceivable that the service had a selection bias that did not accurately reflect the target population and recruited PABC that are more active than the ‘average’ patient. Also, it is likely that HCPs only referred patients they knew would be capable of engaging in physical activity safely. Due to a lack of time and capacity, HCPs make subjective judgements and only refer patients they deem suitable [51].

### Diversity of referrals

The high level of education suggests that PABC referred to the AE service were of high socioeconomic status. People from a higher socioeconomic status participate in more exercise than those with lower socioeconomic status [67]. It appears that the AE project, as with traditional exercise referral schemes, reproduced the inequality paradox whereby services may be successful at the population level but exacerbate existing inequalities by benefiting more affluent groups than less affluent ones [68].

Additionally, service users were majority White British. The service received few referrals from ethnic minority groups. In Sheffield, 13% of people are from ethnic minority communities [69]. Therefore, the needs of these populations are not being fulfilled. Innovations are required to help recruit, engage, retain, and promote health among diverse communities [70]. Previous research has suggested adapting programmes to tailor the design to the needs of a particular cultural group [71]. Future services should investigate if a routine referral for people affected by cancer, rather than ad hoc referrals, improves the diversity of the service users. The Moving Through Cancer agenda aims to ensure that by 2029, all individuals diagnosed with cancer are assessed, advised, referred to, and supported to engage in exercise and rehabilitation programming as the standard of care [72].

### Attrition

Whilst there were benefits from participation in the AE project, attrition from the service was high. Of the 252 people who attended a baseline session, 115 people attended a 3-month follow-up, a loss of 54%. Whilst this drop-out rate appears high, previous research has shown drop-out rates from 80 to 63% at 3 months [73, 74]. AE was a pragmatic service available for anyone affected by cancer who desired PA support. The service aimed to be person-centred so the participants could use it according to their needs.

Many people who did not attend a follow-up appointment stated that the service had helped them begin physical activity, and they no longer needed support. Although this is a positive outcome for the person, it hinders the evaluation of the service. Services must collect why people do not want a follow-up to determine if a service has met their needs. However, the project team found it challenging to monitor and follow-up participants after signposting into city-wide exercise opportunities. Previous research has highlighted the challenges of collecting data on delivering interventions in a field-based setting [73].

## Limitations

Despite the positive outcomes of the AE service, the evaluation highlighted limitations in the service. Reliance on self-report measures enabled the service to be delivered at scale but with limited data integrity and quality. Additionally, the funder determined outcome measures may not have been appropriate. For example, general self-efficacy was measured rather than exercise self-efficacy. Selection bias was observed for individuals referred to the service, with people already engaged and interested in physical activity being referred. Additionally, the service received few referrals for people from ethnic minority communities.

Due to AE being a service, no control group was recruited, limiting the ability of the results to be generalisable to other populations and settings. The programme had a high attrition rate which creates the possibility that the improvements in outcomes are due to the sample remaining in the study. However, the attrition rate was superior to previous research on similar schemes. Additionally, some participants stated that the service had helped them initiate exercise and therefore did not require further support.

## Conclusions

This paper discussed the design, implementation, and evaluation of AE, a person-centred exercise service for PABC, using the Macmillan Cancer Support behaviour change pathway. The service, conducted over 3 years, resulted in positive health and wellbeing outcomes in those who engaged in the service. The results of this evaluation have several implications for the future delivery of community-based exercise services for PABC. Implementing a pathway of care from clinical to community-led care across a city, integrating within existing infrastructure, is complex but has a significant opportunity to transform lives, which the data here infers. However, it is crucial to build upon learning from AE and develop an ecosystem that supports and encourages active behaviour in PABC. Future services must routinely include exercise referrals/prescriptions as a standard part of cancer care [15] to help engage inactive individuals, people from broader ethnic minority groups, and a wider socioeconomic population. More work is needed to increase the knowledge and competencies of stakeholders (HCPS, PABC, and leisure providers). This will make accessing exercise support in the community easier for PABC. Finally, further investment in physical activity pathways could result in a larger picture of national impact.

## Supplementary information

Supplementary file

## Data Availability

N/A

## Code availability

N/A

## References

[1] Ahmad AS, Ormiston-Smith N, Sasieni PD (2015). Trends in the lifetime risk of developing cancer in Great Britain: comparison of risk for those born from 1930 to 1960. Br J Cancer.

[2] Cancer Research UK (2019) Cancer statistics for the UK. Cancer research UK website. Available at: https://www.cancerresearchuk.org/health-professional/cancer-statistics/statistics-by-cancertype#heading-Two. Accessed 16 Feb 2022

[3] Campbell KL, Winters-Stone KM, Wiskemann J, May AM, Schwartz AL, Courneya KS, Zucker DS, Matthews CE, Ligibel JA, Gerber LH, Morris GS, Patel AV, Hue TF, Perna FM, Schmitz KH (2019). Exercise guidelines for cancer survivors : consensus statement from international multidisciplinary roundtable.

[4] Macmillan Cancer Support (2013). Cured – but at what cost?.

[5] Robb K, Transforming cancer services team (2017) Cancer rehabilitation: a scoping report for London. Healthy London Partnership, London

[6] National Cancer Action Team (2013) Cancer rehabilitation making excellent cancer care possible. National Cancer Action Team, London

[7] Asher A, Ng A, Engle J, Gottlieb RA, Mehta PK (2017). Chapter 20 - Principles of cancer rehabilitation. Cardio-oncology.

[8] Cormie P, Atkinson M, Bucci L, Cust A, Eakin E, Hayes S, Mccarthy S, Murnane A, Patchell S, Adams D (2018). Clinical Oncology Society of Australia position statement on exercise in cancer care. Medical Journal of Australia.

[9] Thomas R, Kenfield SA, Yanagisawa Y, Newton RU (2021). Why exercise has a crucial role in cancer prevention, risk reduction and improved outcomes. British Medical Bulletin.

[10] McTiernan A, Friedenreich CM, Katzmarzyk PT, Powell KE, Macko R, Buchner D, Pescatello LS, Bloodgood B, Tennant B, Vaux-Bjerke A, George SM, Troiano RP, Piercy KL (2019). Physical activity in cancer prevention and survival: a systematic review. Medicine & Science in Sports & Exercise.

[11] Turner RR, Steed L, Quirk H, Greasley R, Saxton J, Taylor S, Rosario D, Thaha M, Bourke L (2018). Interventions for promoting habitual exercise in people living with and beyond cancer. Cochrane Database of Systematic Reviews.

[12] Belloni S, Arrigoni C, Caruso R (2021). Effects from physical exercise on reduced cancer-related fatigue: a systematic review of systematic reviews and meta-analysis. Acta Oncologica.

[13] Fukushima T, Nakano J, Hashizume K, Ueno K, Matsuura E, Ikio Y, Ishii S, Morishita S, Tanaka K, Kusuba Y (2021). Effects of aerobic, resistance, and mixed exercises on quality of life in patients with cancer: a systematic review and meta-analysis. Complementary Therapies in Clinical Practice.

[14] Buffart LM, Galvão DA, Brug J, Chinapaw MJM, Newton RU (2014). Evidence-based physical activity guidelines for cancer survivors: current guidelines, knowledge gaps and future research directions. Cancer Treat Rev.

[15] Robinson R, Crank H, Humphreys H, Fisher P, Greenfield DM (2022) Time to embed physical activity within usual care in cancer services: a qualitative study of cancer healthcare professionals’ views at a single centre in England. Disability and Rehabilitation:1–9. 10.1080/09638288.2022.2134468 10.1080/09638288.2022.2134468 36369938

[16] Dempsey PC, Friedenreich CM, Leitzmann MF, Buman MP, Lambert E, Willumsen J, Bull F (2020). Global public health guidelines on physical activity and sedentary behavior for people living with chronic conditions: a call to action. Journal of Physical Activity and Health.

[17] World Health Organisation (2020) WHO guidelines on physical activity and sedentary behaviour. Geneva, Switzerland

[18] Lynch BM, Dunstan DW, Vallance JK, Owen N (2013). Don’t take cancer sitting down. Cancer.

[19] Eng L, Pringle D, Su J, Shen X, Mahler M, Niu C, Charow R, Tiessen K, Lam C, Halytskyy O, Naik H, Hon H, Irwin M, Pat V, Gonos C, Chan C, Villeneuve J, Harland L, Shani RM, Brown MC, Selby P, Howell D, Xu W, Liu G, Alibhai SMH, Jones JM (2018). Patterns, perceptions, and perceived barriers to physical activity in adult cancer survivors. Support Care Cancer.

[20] The Lancet Oncology (2018). Exercise and cancer treatment: balancing patient needs. The Lancet Oncology.

[21] Bourke L, Homer KE, Thaha MA, Steed L, Rosario DJ, Robb KA, Saxton JM, Taylor SJC (2013). Interventions to improve exercise behaviour in sedentary people living with and beyond cancer: a systematic review. Br J Cancer.

[22] Cantwell M, Walsh DMJ, Furlong B, Moyna N, McCaffrey N, Woods C (2020). The development of the MedEx IMPACT intervention: a patient-centered, evidenced-based and theoretically-informed physical activity behavior change intervention for individuals living with and beyond cancer. Cancer Control.

[23] Schmitz KH, Campbell AM, Stuiver MM, Pinto BM, Schwartz AL, Morris GS, Ligibel JA, Cheville A, Galvão DA, Alfano CM, Patel AV, Hue T, Gerber LH, Sallis R, Gusani NJ, Stout NL, Chan L, Flowers F, Doyle C, Helmrich S, Bain W, Sokolof J, Winters-Stone KM, Campbell KL, Matthews CE (2019). Exercise is medicine in oncology: engaging clinicians to help patients move through cancer. CA: A Cancer Journal for Clinicians.

[24] Morris M, Crank H, Loosemore M, Stevinson C (2020). Identification of research priorities in exercise oncology: a consensus study. Journal of Cancer.

[25] HM Government (2015) Sporting future: a new strategy for an Active Nation. Sporting Future, London

[26] Moreton R, Stutz A, Robinson S, Mulla I, Winter M, Roberts J, Hillsdon M (2018) Evaluation of the Macmillan physical activity behaviour change care pathway Final report 2018. CFE

[27] Humphreys L, Crank H, Frith G, Speake H, Reece LJ (2019). Bright spots, physical activity investments that work: Active Everyday, Sheffield’s physical activity service for all people living with and beyond cancer. British Journal of Sports Medicine.

[28] Foster J, Worbey S, Chamberlain K, Horlock R, Marsh T (2018) Integrating physical activity into cancer care: evidence and guidance. Macmillan Cancer Support

[29] Foster J, Thompson K, Harkin J (2012) Let’s Get Moving – A physical activity care pathway. Commissioning Guidance. Department of Health/ Physical Activity Policy, London

[30] National Institute for Health and Care Excellence (2014) Behaviour change: individual approaches. NICE Public Health Guidance 49 (PH49). NICE, London

[31] Rowe J, Young N, Rowlands S (2014) The recovery package: sharing good practice. Macmillan Cancer Support

[32] Humphreys L, Crank H, Dixey J, Greenfield DM (2020). An integrated model of exercise support for people affected by cancer: consensus through scoping. Disability and Rehabilitation.

[33] Miller WR, Rollnick S (2013). Motivational interviewing: helping people change.

[34] Macmillan Cancer Support (2013) The cancer and physical activity standard evaluation framework. Available from: https://www.macmillan.org.uk/documents/aboutus/health_professionals/physicalactivity/cancer-physical-activity-standard-evaluation-framework-measurement-tools.pdf

[35] Lowther M, Mutrie N, Loughlan C, Mcfarlane C (1999). Development of a Scottish physical activity questionnaire: a tool for use in physical activity interventions. Br J Sports Med.

[36] Cella D, Lai J, Stone A (2011). Self-reported fatigue: one dimension or more? Lessons from the Functional Assessment of Chronic Illness Therapy-Fatigue (FACIT-F) questionnaire. Support Care Cancer.

[37] Schwarzer R, Jerusalem M (1995). Generalised self-efficacy scale. Anonymous measures in health psychology: a user’s porfolio.

[38] EuroQol (1990). EuroQol - a new facility for the measurement of health-related quality of life. Health Policy.

[39] Wright Foundation Trust (2021) Level 4 cancer rehabilitation. WRIGHT Foundation CIC. Available at: https://www.wrightfoundation.com/course/level-4-cancer-rehabilitation. Accessed 15 Oct 2022

[40] CanRehab (2022) Training in cancer and exercise rehabilitation. Available at: www.canrehab.com/professions/fitness. Accessed 18 Nov 2022

[41] National Cancer Registration and Analysis Service (NCRAS)(2019) Cancer data. Available from: https://www.cancerdata.nhs.uk/. Accessed 15 Feb 2022

[42] National Cancer Registration and Analysis Service (2019) Cancer data. https://www.cancerdata.nhs.uk/dashboard#?tab=Overview. Accessed 01/07/ 2022.

[43] Queen M, Karatzaferi C, Bloxham SR, Panwar U, Drew P, Barton AG, Edwards AM, Sakkas GK (2016). How can physical activity referral rates for breast cancer patients be increased?. Front Oncol.

[44] Din NU, Moore GF, Murphy S, Wilkinson C, Williams NH (2015). Health professionals’ perspectives on exercise referral and physical activity promotion in primary care: findings from a process evaluation of the National Exercise Referral Scheme in Wales. Health Educ J.

[45] Foster J, Horlock R, Worbey S (2018) Physical activity and cancer: the underrated wonder drug. The case for integrating physical activity into cancer care. Macmillan Cancer Support

[46] Williams K, Beeken RJ, Wardle J (2013). Health behaviour advice to cancer patients: the perspective of social network members. British Journal of Cancer.

[47] Haussmann A, Ungar N, Tsiouris A, Depenbusch J, Sieverding M, Wiskemann J, Steindorf K (2021). Physical activity counseling to cancer patients: how are patients addressed and who benefits most?. Patient Education and Counseling.

[48] Joseph R, Hart NH, Bradford N, Agbejule OA, Koczwara B, Chan A, Wallen MP, Chan RJ (2022). Diet and exercise advice and referrals for cancer survivors: an integrative review of medical and nursing perspectives. Support Care Cancer.

[49] Nadler M, Bainbridge D, Tomasone J, Cheifetz O, Juergens RA, Sussman J (2017). Oncology care provider perspectives on exercise promotion in people with cancer: an examination of knowledge, practices, barriers, and facilitators. Support Care Cancer.

[50] Roberts AL, Potts HWW, Stevens C, Lally P, Smith L, Fisher A (2019). Cancer specialist nurses’ perspectives of physical activity promotion and the potential role of physical activity apps in cancer care. J Cancer Surviv.

[51] Speake H, Copeland RJ, Till SH, Breckon JD, Haake S, Hart O (2016). Embedding physical activity in the heart of the NHS: the need for a whole-system approach. Sports Medicine.

[52] Pulvirenti M, Mcmillan J, Lawn S (2014). Empowerment, patient centred care and self-management. Health Expectations.

[53] Santa Mina D, Sabiston CM, Au D, Fong ‡AJ, Capozzi Md Phd LC, Langelier D, Chasen M, Chiarotto J, Tomasone JR, Jones JM, Chang ‡E, Culos-Reed Phd § † †, S. N. (2018) Connecting people with cancer to physical activity and exercise programs: a pathway to create accessibility and engagement. Current Oncology:25, 149–162. 10.3747/co.25.3977 10.3747/co.25.3977 PMC5927786 29719431

[54] Macmillan Cancer Support (2016) What motivates people with cancer to get active:? Understanding the motivations and barriers to physical activity in people living with cancer

[55] Granger CL, Parry SM, Edbrooke L, Abo S, Leggett N, Dwyer M, Denehy L (2018). Improving the delivery of physical activity services in lung cancer: a qualitative representation of the patient’s perspective. Eur J Cancer Care.

[56] Murray J, Perry R, Pontifex E, Selva-Nayagam S, Bezak E, Bennett H (2022). The impact of breast cancer on fears of exercise and exercise identity. Patient Education and Counseling.

[57] Stout NL, Brown JC, Schwartz AL, Marshall TF, Campbell AM, Nekhlyudov L, Zucker DS, Basen-engquist KM, Campbell GM, Meyerhardt JJ, Cheville AL, Covington KR, Ligibel JA, Sokolof JM, Schmitz KH, Alfano CM (2020). An exercise oncology clinical pathway: screening and referral for personalized interventions. Cancer.

[58] Chan A, Ports K, Neo P, Ramalingam MB, Lim AT, Tan B, Hart NH, Chan RJ, Loh K (2022). Barriers and facilitators to exercise among adult cancer survivors in Singapore. Supportive Care in Cancer.

[59] Hefferon K, Murphy H, McLeod J, Mutrie N, Campbell A (2013). Understanding barriers to exercise implementation 5-year post-breast cancer diagnosis: a large-scale qualitative study. Health Education Research.

[60] Hardcastle SJ, Maxwell-Smith C, Kamarova S, Lamb S, Millar L, Cohen PA (2018). Factors influencing non-participation in an exercise program and attitudes towards physical activity amongst cancer survivors. Supportive Care in Cancer.

[61] Kuchinski A, Ayhan MR, Lash A (2009). Treatment-related fatigue and exercise in patients with cancer: a systematic review. Medsurg Nurs.

[62] Schellekens MPJ, Wolvers MDJ, Schroevers MJ, Bootsma TI, Cramer AOJ, van der Lee ML (2019). Exploring the interconnectedness of fatigue, depression, anxiety and potential risk and protective factors in cancer patients: a network approach. J Behav Med.

[63] Whitehead BR, Blaxton JM (2021). Daily associations among aging perceptions, perceived health, and perceived stress in older adults. Aging & Mental Health.

[64] Williams NH, Hendry M, France B, Lewis R, Wilkinson C (2007). Effectiveness of exercise-referral schemes to promote physical activity in adults: systematic review. British Journal of General Practice.

[65] Ng AH, Ngo-Huang A, Vidal M, Reyes-Garcia A, Liu DD, Williams JL, Fu JB, Yadav R, Bruera E (2021). Exercise barriers and adherence to recommendations in patients with cancer. JCO Oncology Practice.

[66] Bulley C, Donaghy M, Payne A, Mutrie N, Margaret Q (2005). Validation and modification of the Scottish Physical Activity Questionnaire for use in a female student population. International Journal of Health Promotion and Education.

[67] Tucker-Seeley R, Subramanian SV, Li Y, Sorensen G (2009). Neighborhood safety, socioeconomic status, and physical activity in older adults. Am J Prev Med.

[68] Williams O, Gibson K (2018). Exercise as a poisoned elixir: inactivity. inequality and intervention.

[69] Gilbertson J, Dayson C, Leather D (2020) An evaluation of the impact of Weston Park Cancer Support Centre. 10.7190/cresr.2019.3247799239

[70] Murray KE, Ermias A, Lung A, Mohamed AS, Ellis BH, Linke S, Kerr J, Bowen DJ, Marcus BH (2017). Culturally adapting a physical activity intervention for Somali women: the need for theory and innovation to promote equity. Translational Behavioral Medicine.

[71] Castro FG, Barrera M, Holleran Steiker LK (2010). Issues and challenges in the design of culturally adapted evidence-based interventions. Annual Review of Clinical Psychology.

[72] Schmitz KH, Stout NL, Maitin-Shepard M, Campbell A, Schwartz AL, Grimmett C, Meyerhardt JA, Sokolof JM (2021). Moving through cancer: setting the agenda to make exercise standard in oncology practice. Cancer.

[73] Bull FC, Milton KE (2010). A process evaluation of a “physical activity pathway” in the primary care setting. BMC Public Health.

[74] McGeechan GJ, Phillips D, Wilson L, Whittaker VJ, O’Neill G, Newbury-Birch D (2017). Service evaluation of an exercise on referral scheme for adults with existing health conditions in the United Kingdom. Int J Behav Med.

